# Seventy-Day Toxicity Study in Juvenile Sprague-Dawley Rats with Semicarbazide (SEM) from Weaning to Sexual Maturity

**DOI:** 10.1155/2022/5059761

**Published:** 2022-07-22

**Authors:** Vijaykumar Malashetty, Raghunandan Deshpande, Somnathreddy Patil

**Affiliations:** ^1^Reproductive Biology and Mechanistic Toxicology Research Laboratory, Department of Studies in Zoology, Vijayanagara Sri Krishnadevaraya University, Ballari 583105, India; ^2^H.K.E.S's Matoshree Taradevi Rampure Institute of Pharmaceutical Sciences, Kalaburagi 585105, India; ^3^Department of P.G. Studies and Research in Zoology, Government College, Kalaburagi 85105, India

## Abstract

The study was conducted to evaluate the toxicological effects, functional observation battery tests, and sexual maturity of semicarbazide oral gavage administration to juvenile Sprague-Dawley rats for 70 days at 0, 15, 30, and 60 mg/kg/day weaning to sexual maturity. At 60 mg/kg/day, there was a delay in mean age at acquisition of balano-preputial and vaginal patency and a decrease in body weight and food consumption in males. Treatment increased reticulocyte count, aspartate aminotransferase, and alanine aminotransferase levels in both sexes and decreased hematocrit and protein in males. Increased absolute and relative liver and spleen weight in both sexes were observed. Male rats had lower thymus and testes weights, whereas female rats had lower uterine weights. Semicarbazide caused significant changes in sperm motility, sperm count, and sperm abnormality. Histopathologically, semicarbazide caused cortical hypertrophy in adrenals and increased extramedullary hematopoiesis in the spleen; hepatocellular hypertrophy, follicular epithelial hypertrophy in the thyroid, and degeneration of seminiferous tubules in the testis were observed at 60 mg/kg/day when compared to control. Results suggest that 60 mg/kg/day of semicarbazide can exert systemic toxicity in juvenile rats. The no observed adverse effect level (NOAEL) of semicarbazide for juvenile Sprague-Dawley rats was estimated to be 30 mg/kg/day.

## 1. Introduction

The semicarbazide (SEM) is the raw material of semicarbazone and has been shown to possess antifungal and antibacterial activities [1, 2] and recent literature showed anticonvulsant [3], antitubercular [4], antioxidant [5], antimicrobial, analgesic, antipyretic [6], and anti-inflammatory activity [7].

SEM showed toxicity in the bone, cartilage, and the aorta in Wistar Hannover GALAS rats [8]. *In vitro*, SEM has been shown to have poor mutagenic activity, but *in vivo* experimental evidence is still insufficient to make a conclusion [9]. In susceptible mouse strains, SEM tends to be a weak carcinogen, but not in rats [10]. SEM is classified by the International Agency for Research on Cancer (IARC) for its carcinogenicity to humans based on partial evidence of carcinogenicity in laboratory animals and there are no published data on humans [11]. Recent data showed marked alterations of spontaneous motor and exploratory behaviors and histological alterations in the thyroid and ovary at 40 mg/kg [12]. Ramos et al. [13] showed SEM affects the testicular morphology of juvenile Wistar rats. In addition, teratogenic effects such as cleft palate, and aortic aneurysms have been reported [14].

Semicarbazide (SEM) is a by-product of azodicarbonamide (ADC), which is found in foods sold in glass jars and bottles with metal lids sealed with plastic gaskets, according to the European Food Safety Authority [15]. SEM is released during manufacture when packaged foods are heated to ensure a tight seal and it is present as a food contaminant in baby foods, fruit juices, jams, and conserves. Moreover, SEM was also found in different foodstuffs such as powder and liquid milk, egg, and whey powder [12, 16, 17]. Since August 2005 (Official Journal of the European Union Commission Directive 2004/1/EC), the European Commission has banned the use of azodicarbonamide (ADC) as a blowing agent and seeking more information on the toxicological impact of SEM. However, ADC is still used in some countries, and this may explain the presence of SEM in a wide range of breaded food products [18]. SEM has also been detected in seaweed-derived products, which are widely used as food additives [19]. While EFSA has stated that the risks to consumers appear to be low, infants are more likely to be exposed to SEM due to their large consumption of products in glass jars. Indeed, the European Food Safety Authority (EFSA) reviewed that SEM levels reported in these foods are variable, up to 25 *µ*g/kg (=25 ppb) but found in higher concentrations in baby foods, possibly because of the higher ratio of the gasket area of food mass for these small pack sizes [20]. Different levels of SEM were measured in a recent European baby food survey, where the values ranged between 5.6 ± 3.8 *µ*g/kg and 53.9 ± 3.1 *µ*g/kg, depending on the type of food within the glass jars [21]. The hazard of adverse effects of SEM exposure to humans has been taken into consideration low because there is a sufficient margin of exposure [19]. However, intake of SEM for infants is estimated to be much above for adults due to high consumption of baby food in glass jars and the infants' small body weight [12]. Accordingly, because infants or children could be more susceptible to SEM than adults, evaluation of toxic effects of SEM with the target on age-related susceptibility is vital for risk assessment in human health.

Taking into account that children can represent a subpopulation with particularly high exposure to SEM, the present study is aimed to evaluate the detailed toxicological effects, Functional Observation Battery (FOB) tests, estrous cyclicity, and sexual maturity of SEM upon oral gavage administration during the most appropriate window of exposure—the juvenile period—namely, from weaning to sexual maturity, in male and female SD rats. Potential target organs and/or tissues have been selected on the basis of the juvenile test on rodents and the literature data on SEM toxicity [12, 22, 23].

## 2. Materials and Methods

### 2.1. Chemicals

SEM was purchased from Sigma–Aldrich (Bengaluru, India).

### 2.2. Test Animals

In-house breed offspring along with dams were acclimatized on PND 18–21 for 5 days before the start of the treatment. The offspring were weaned on PND 21 from dams. A total of 40 male and 40 female juvenile Sprague-Dawley (SD) rats weighing 40–55 g males and 36–53 g females (23–26 days old) were divided into four groups (10 rats in each group) by body weight stratification. Twelve dams were used to get 40 male and 40 female juvenile rats. The weight variation did not exceed ±20% of the mean weight for each sex. The animal experiment was performed in accordance with the guideline for the care and use of laboratory animals [24]. The Institutional Animal Ethics Committee gave its approval to all of the animal experiments.

### 2.3. Housing, Bedding, Diet, and Water

Two juvenile rats of the same sex per cage were housed in sterilized Polysulfone rat cages with paddy husk as bedding material. Rats were housed in a controlled environment room (temperature: 24 ± 2°C with relative humidity 60 and 66%; 12 hours light and 12 hours dark cycle). Pelleted rodent feed (purchased from Hindustan Animal Feeds, Gujarat, India) and purified water in polycarbonate bottles were provided *ad libitum*.

### 2.4. Chemical Preparation

SEM is a crystalline white powder stored under ambient conditions. Daily fresh SEM formulation is prepared by dissolving in Milli-Q water for dose administration.

### 2.5. Treatment

The treatment started from between PND 23 and 26 years, which covers an equivalent human age of approximately 2 years of age [25]. SEM formulation was administered to the rats by oral gavage using the soft-tipped polypropylene flexible plastic tubing (Instech Laboratories) up to the week 5 age and after that curved stainless steel oral gavage needles were used till the end of the treatment period to the specific treatment groups of rats once daily at the same time (varied by ±2 h) for 70 consecutively. Similarly, the control animals were administered an equivalent volume of Milli-Q water. Individual doses were determined based on the most recent weekly body weight and were adjusted weekly to sustain the target dose for all rats (i.e., mg/kg/day). All doses were administered volumetrically at 10 mL/kg. The control group animals received the vehicle (Milli-Q water) only, at the same dose volume as the test animals. For all animals, dose administration was daily for a period of 70 days, which covers the effect of semicarbazide on growth, target organs, neurobehavior, and sexual development. This study was conducted according to (ICH), M3 (R2), 2009 [26].

The selected male and female juvenile rats were assigned to control and treatment groups as given in Table 1.

Three dose levels of 15, 30, and 60 mg/kg/day were selected based on the results of the in-house 14-day oral dose range finding toxicity study with SEM in juvenile SD rats and available literature [12, 27]. In the 14-day dose range finding toxicity study, mortality, transient slight salivation, and reduction in body weight and food consumption was observed in SEM-treated rats at a high dose of 130 mg/kg/day. So the mid-dose of 60 mg/kg/day was considered as a high dose in the present study.

### 2.6. Clinical Signs, Mortality, Detailed Clinical Examination, and Ophthalmological Examination

All rats were observed for clinical signs and mortalities twice a day. Detailed clinical examination was done prior to the test item administration on Day 1 for all animals and at weekly intervals. During the comprehensive clinical examination, all rats were observed for changes in the skin, fur, eyes, mucous membranes, the occurrence of secretions and excretions and autonomic activity (e.g., piloerection, lacrimation, unusual respiratory pattern, pupil size), posture, changes in gait and response to handling, the presence of clonic or tonic movements, and stereotypies (e.g., excessive grooming, repetitive circling, or bizarre behavior (e.g., self-mutilation and walking backward)).

Ophthalmological examination was performed with an ophthalmoscope once before the start of the treatment period and at the end of treatment. Mydriasis was induced before examination using a solution of 1% Tropicamide solution.

### 2.7. Functional Observation Battery Tests

The following neurological examination was conducted during the last week (10th week) of the treatment period for all rats.

### 2.8. Home Cage Observations

All animals were observed in the home cage for posture and for the presence or absence of unusual vocalizations and convulsions.

### 2.9. Open Field Observations

Rats were subjected to an open-field test. The rats were placed in an open-field arena with a clean absorbent paper and observed for 2 min. The following behavioral parameters were scored: gait, posture, mobility score, arousal level, clonic or tonic movements, stereotypic behaviors, bizarre behavior, urination, defecation, rearing, and vocalizations [28].

### 2.10. Sensory Reactivity Measurements

During sensory reactivity measurements, rats were observed and recorded for the following observations: approach response, touch response, click response, tail-pinch response, pupil response, and aerial righting reflex [28–31].

### 2.11. Motor Activity

An electronic animal activity measuring device (Columbus Instruments) was used to monitor the motor activity of rats. Each rat was placed individually within the activity cages of the instrument. The rats were monitored for 60 min. During this motor activity following parameters were measured: the stereotypic time (small movements) in seconds, the ambulatory time (large ambulatory movement) in seconds, horizontal counts, and ambulatory counts [32].

### 2.12. Hindlimb Landing Foot Splay

The landing foot splay was assessed using a similar procedure as published by Edwards and Parker [33]. The heel portion of the hindfoot of each animal was marked with ink and the animal was then dropped from a height of 30 cm onto a recording sheet. This procedure was repeated three times. The distance from the center to the center of the inking marks was measured in centimeters (cm), and the mean of the three splash values was used for the statistical analysis.

### 2.13. Grip Performance

The grip performance of the hind and forelimbs was tested using a computerized dual gripping force measuring device (make: Orchid Scientific). Three trials were performed for each rat, i.e., three trials each for forelimbs and hindlimbs. The average of three trials for both forelimbs and hindlimbs was calculated [34].

### 2.14. Body Weight and Food Consumption

Individual body weights were recorded on Day 1 of treatment (prior to treatment) and approximately weekly thereafter (intervals of 7 days ± 1). The food consumption was measured at weekly intervals, and the individual food consumption was calculated by using the food consumed at each measuring interval and the number of days in the intervening period to determine the food intake/day.

### 2.15. Estrous Cyclicity

The estrous cycle length and pattern were evaluated by vaginal smears, and the stage of the estrous cycle was recorded daily for two weeks prior to necropsy. The length of the estrous cycle was computed for all females as the period between two successive diestrus stages.

### 2.16. Indices of Sexual Maturation

The age and body weight at the vaginal opening and preputial separation was recorded [35].

### 2.17. Clinical Pathology Investigations

Clinical pathology was performed on all animals (Groups 1–4) for blood chemistry and hematology (Day 71). The animals were fasting the entire night before the blood sample. Blood samples were collected by retro-orbital sinus puncture under isoflurane anesthesia prior to terminal sacrifice. Blood was collected into K2EDTA and heparinized tubes for analysis of hematology and clinical chemistry parameters, respectively. The following hematological parameters were determined using ADVIA 2120 hematology system: Hb, RBC, WBC, Plat, Hct, MCV, MCH, MCHC, DLC, Retic, and MPV. The following clinical parameters were determined using Dimension RxL MaX Clinical Chemistry System: ALT, Alb, ALP, AST, BUN, Creat, GGT, Glu, Pi, K, Na, LDH, T. Chol, T. Pro, T. Bil, Trig, Glob, A/G, Ca, Cl.

TSH and T4 were estimated by the Enzyme-Linked Immunosorbent Assay (ELISA) method (Endocrine Technologies Inc., USA) for the samples.

### 2.18. Sperm Evaluation

At necropsy, the right epididymis was collected and frozen for sperm count and the right vas deferens was collected for evaluation of sperm motility and sperm morphology. Sperm motility was evaluated using SCA® (Sperm Class Analyzer) CASA (computer-aided sperm analysis) system (SCA Microptic SL, Barcelona, Spain).

### 2.19. Organ Weights and Histopathology

At terminal sacrifice, all animals (Groups 1–4) were euthanized by exsanguination under isoflurane anesthesia after blood collection for clinical pathology. The brain, coagulating glands with seminal vesicles, epididymides, heart, kidneys, liver, ovaries, pituitary gland, prostate, spleen, testes, thymus, thyroid and parathyroid, and uterus with the cervix were weighed. The following organs and tissues from all animals were preserved in 10% neutral buffered formalin for histopathological examination: adrenals, bone marrow smear (from femur), brain (cerebrum, cerebellum, and medulla), bulbourethral gland, cecum, coagulating glands with seminal vesicles, colon, diaphragm, duodenum, femur with joint, heart, harderian glands, ileum (with Peyer's patches), jejunum, kidneys, liver, lungs, mammary glands, mandibular lymph nodes, mesenteric lymph nodes, ovaries, pancreas, pituitary gland, preputial gland, prostate, rectum, salivary glands (sublingual, mandibular, parotid), sciatic nerves, spleen, spinal cord (cervical, thoracic and lumbar), sternum with marrow, stomach, thymus, trachea, thyroid and parathyroid, uterus with the cervix, and urinary bladder. The testes and epididymides were collected in modified Davidson's fluid. The eyes were collected in Davidson's fluid.

The tissues were processed for routine paraffin embedding and 4-5 micron sections using a Leica® microtome and mounted on slides, which were subsequently stained with Mayer's Hematoxylin and Eosin.

### 2.20. Statistical Analysis

Differences between groups were determined by one-way analysis of variance (ANOVA) using Statistical Package for Social Sciences (SPSS, version 20.0) software for windows and post hoc test for intergroup using the least significant difference, followed by Dunnett's test. Significance was considered at *p* < 0.05. All results were expressed as the mean ± standard deviation of the mean.

## 3. Results

### 3.1. Clinical Signs, Mortality, Detailed Clinical Examination, and Ophthalmological Examination

No SEM-related clinical signs and mortality were observed in the animals throughout the experiment. However, a clinical sign of sparse hair loss at the right and left flank was observed in a female of the control group and one male rat of the high dose group was considered a spontaneous finding and not related to treatment. However, incidental slight alopecia of the head and trunk in 2/10 in 60 mg/kg/day males and slight to moderate alopecia of the head and hindlimb in 1/10 in 30 mg/kg/day females were considered spontaneous finding and not related to treatment.

An ophthalmological examination did not reveal any eye abnormalities.

### 3.2. Functional Observation Battery

#### 3.2.1. Home Cage, Open Field Observations, and Sensory Reactivity Measurements

No treatment-related abnormalities were observed in the home cage and open-field observations at all the treated dose levels in either sex (Table 2).

#### 3.2.2. Motor Activity

Automated motor activity (Table 3) assessments of animals showed no significant changes in SEM-treated animals compared to concurrent controls.

#### 3.2.3. Hindlimb Landing Foot Splay and Grip Performance

There were no treatment-related significant variations observed in hindlimb foot splay and grip performance in either sex when compared to concurrent controls (Table 3).

### 3.3. Body Weight and Food Consumption

**Figure 1:**
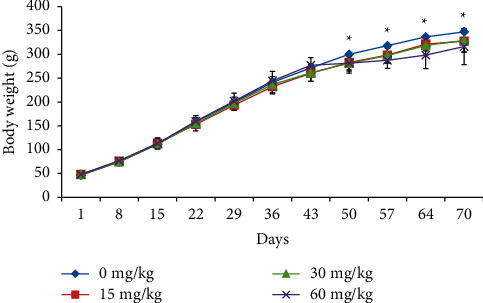
Body weight of male rats treated with SEM.

**Figure 2:**
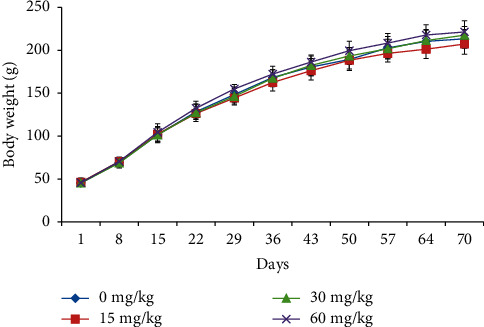
Body weight of female rats treated with SEM.

A statistically significant decreased body weight was observed in male rats at 60 mg/kg during treatment days 50–70 (Figure 1). However, in female rats, no significant change in the body weight was observed at any doses when compared to control rats (Figure 2).

**Figure 3:**
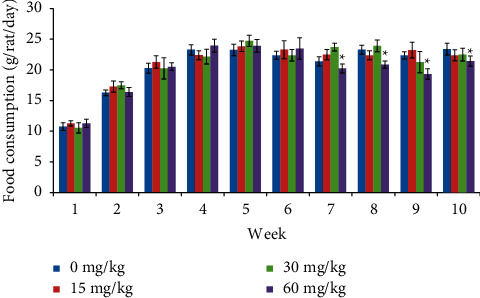
Food consumption of male rats treated with SEM.

**Figure 4:**
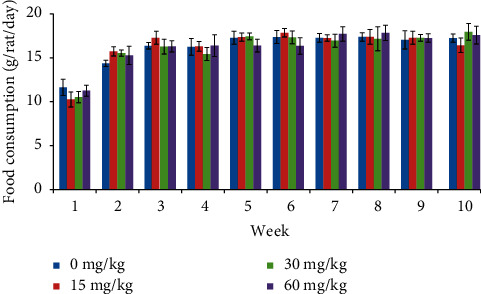
Food consumption of female rats treated with SEM.

Food consumption was significantly decreased (Figure 3) in males at 60 mg/kg from week 7 and continued till the end of the treatment period. In females, food consumption was comparable with the control animals throughout the study period (Figure 4).

### 3.4. Estrous Cyclicity

The calculated mean estrous cycle length was 4.76, 4.43, 4.58, and 4.32 days in vehicle control, 15, 30, and 60 mg/kg/day doses, respectively. The mean estrous cycle length in the treated groups was not significantly different from the vehicle control group (Table 4).

### 3.5. Indices of Sexual Maturation

When soft pressure is applied to the animal's prepuce, the prepuce fully retracts from the penis. The average age at acquisition of balano-preputial separation in the control, 15, 30, and 60 mg/kg/day exposure groups were 45.98 ± 1.07, 46.88 ± 2.11, 47.63 ± 1.63, and 48.28 ± 1.94 days, respectively. The mean age at acquisition of balano-preputial was statistically higher in the 30 and 60 mg/kg/day dose group (Table 5).

The noticeable split in the membranous sheath covering the vaginal orifice that causes the vaginal edges to separate is referred to as a vaginal opening. The average duration post weaning of vaginal opening (patency) in the control, 15, 30, and 60 mg/kg/day exposure groups were 36.21 ± 1.26, 36.89 ± 1.13, 37.27 ± 1.2, and 39.11 ± 1.13 days, respectively. The mean age at acquisition of vaginal patency was significantly delayed by 3 days at 60 mg/kg/day when compared to the vehicle control group (Table 5).

Body weights at the age of preputial separation and vaginal opening are shown in Table 5 and were significantly increased in the treated groups as compared with the control.

### 3.6. Clinical Pathology Investigations

#### 3.6.1. Hematology Parameters

An increase in reticulocyte count was observed in both sexes at 60 mg/kg/day.

This finding was associated with increased hematopoiesis and the presence of megakaryocytes in spleen, microscopically and was not accompanied by any change in other RBC-related parameters.

In males, Hct decreased at 60 mg/kg/day group compared with the vehicle control group. No significant differences in other hematological parameters of the 15, 30, and 60 mg/kg/day treated rats as compared with those of rats in the normal control group (Table 6).

#### 3.6.2. Clinical Chemistry

Total protein level decreased in males in the 60 mg/kg/day groups compared with in the control groups. AST and ALT levels were significantly increased in both the sexes at the 60 mg/kg/day group (Table 7).

There were no test item-related changes observed in thyroid stimulating hormone (TSH) and thyroxin hormone (T4) levels in adult males and females at termination (Table 8).

### 3.7. Organ Weights

The majority of the tissues examined showed no changes in their absolute and relative weights against body weight when compared to concurrent controls. The absolute and relative weights of the liver and spleen were significantly increased in both the sexes at the 60 mg/kg/day group. However, the absolute and relative weight of the testes and thymus were significantly decreased in males, and a marginal decrease in the absolute and relative weight of uterus with cervix was decreased in females at 60 mg/kg/day group (Tables 9 and 10).

### 3.8. Sperm Evaluation

Significant decreases in the values for percent progressive motile sperms and percent motile sperms were recorded in the 60 mg/kg/day group. Significant decrease in the percent of normal sperms and an increased percentage of abnormal sperms such as giant heads, round heads, tailless heads, and headless tails were recorded at 60 mg/kg/day dose group, compared to the vehicle control. The commonly observed abnormalities across groups were headless or tailless sperms. The values for cauda epididymis weight, number of sperms per cauda epididymis, and number of sperms per gram of cauda epididymis did not differ significantly among the control and high dose group rats (Table 11).

### 3.9. Histopathology

The histopathological evaluations of the selected organs did not reveal any morphological abnormalities that could be attributed to the administration of SEM to the rats. However, following microscopic changes were observed in 60 mg/kg/day group animals.

Increased extramedullary hematopoiesis in the spleen, cortical hypertrophy in adrenals involving zona fasciculata of adrenals in males, and both zona fasciculata and zona glomerulosa in females were observed. The lymphoid depletion in the thymus was observed. A minimal degree of centrilobular hepatocyte hypertrophy in both males and females was observed (Table 12).

## 4. Discussion and Conclusion

The main objective of the present study was to comprehensively evaluate the toxicity of SEM in juvenile Sprague-Dawley rats exposed orally for 70 days from weaning to sexual maturation. Few reports have been published on the subchronic oral toxicity of SEM. However, in the present study, mild hepatotoxicity and sperm parameters were affected, which was not reported previously. The rate of mortality, general behavioral changes, and body weight are preliminary indicators of early signs of toxicity caused by various chemicals and drugs [36, 37]. During the experimental period, no mortality and clinical signs were noticed at 60 mg/kg/day in any of the treated groups of either sex. However, previous reports showed mortality at 40 and 75 mg/kg/day [12]. The decreased body weight in males correlated with the decreased food consumption during the period and the data are consistent with previous work reported by other authors [12, 13]. FOB is a non-invasive procedure developed to detect gross functional deficits in young adult rats after they have been exposed to drugs or chemicals. This battery of tests does not include a comprehensive assessment of neurotoxicity, although it can be used in combination with neuropathologic assessment and/or general toxicity testing to provide a more complete picture [38, 39]. In our FOB tests, no abnormalities were found in the SEM-treated rats when compared with the control.

In humans and animals, the hematopoietic pathway is one of the most critical targets for toxic substances and a significant indicator of physiological and pathological states [40]. An increase in reticulocyte count was observed in both sexes at 60 mg/kg/day. This finding was associated with increased hematopoiesis and the presence of megakaryocytes in the spleen, microscopically and was not accompanied by any change in other RBC-related parameters. In males, Hct decreased at 60 mg/kg/day group compared with the vehicle control group. Slight variations in mean values, as is common for high precision measurements, can be statistically significant but are not toxicologically significant.

Since both the liver and the kidney are needed for an organism's survival, biochemical parameters are considered an appropriate predictor for toxicity evaluation [41]. In addition, when the membrane of a liver cell is weakened, a number of enzymes usually found in the cytosol are released into the bloodstream. The ability to evaluate liver function by measuring the activities of serum marker enzymes such as ALT, AST, and ALP, as well as the amount of serum total bilirubin, has proven to be a useful tool [42]. It is common with hepatocellular hypertrophy to get some increase in ALT or AST with no evidence of hepatic injury, therefore increase should not be considered adverse until they are at least 2-fold to 3-fold greater than control levels. Below that level, they are usually within the normal range for the animal [43]. In the present study, the increased AST and ALT are 14% and 21% in males and 15% and 36% in females, respectively, indicating that the SEM is mild hepatotoxic in rats and considered to be non-adverse and these values are reversible after at the end of 14 day recovery period (data not shown).

In the developing male reproductive tract, SEM seems to exert subtle adverse effects by altering the percentage of testicular tissue programmed for spermatogenesis without affecting spermatogenesis itself [12]. In the present study, a significantly decreased number of sperms, percentage of progressive motile sperms and motile sperms, and also an increase in the percentage of abnormal sperms were observed. However, Maranghi et al. [12] observed only subtle adverse effects of SEM in the testis of juvenile Sprague-Dawley rats by altering the percentage of testicular tissue programmed for spermatogenesis without affecting spermatogenesis itself and sperm data are contradictory.

Increased extramedullary hematopoiesis in the spleen was correlated with increased reticulocytes count, cortical hypertrophy in adrenals this involves zona fasciculata of adrenals in males and both zona fasciculata and zona glomerulosa in females was observed. Hepatocellular hypertrophy in both sexes was observed.

After neonatal age, the thymus is required for proper ovarian growth and function, and abnormal thymus function may result in delayed puberty and altered uterus weight [44, 45]. The lymphoid depletion in the thymus and delayed puberty (vaginal patency and preputial separation) were observed. The vaginal opening and the first ovulation are decisive markers of puberty in female rats [46, 47]. In the present study, the days of vaginal opening were postponed by SEM treatment, suggesting that prepubertal exposure to SEM can delay the onset of puberty. Preputial separation in untreated rats initiates from cornification of the epithelium lying between the glans penis and prepuce [48, 49]. It is known that lower body weight in both humans and laboratory animals will delay the onset of puberty and reduce fertility in adults [50]. The age of vaginal opening in females and preputial separation in males observed in this study was not totally dependent upon the body weight. Some of the animals with lower body weights reached the vaginal opening and preputial separation before others with greater body weights. Since we did not include any reversibility data of SEM at the doses administered (15, 30, and 60 mg/kg/day) in this present study.

Therefore, overall the results of the present study indicate that the NOAEL in juvenile rats is 30 mg/kg/day for SEM oral gavage administration.

## Figures and Tables

**Table 1:** Experimental design.

Group	Target dose level (mg/kg/day)	Concentration (mg/mL)	Dose volume (mL/kg)	No. of animals/group (M/F)
1	Vehicle control	0	10	10/10
2	15, low dose	1.5	10	10/10
3	30, mid dose	3	10	10/10
4	60, high dose	6	10	10/10

**Table 2:** Functional observation battery in male and female rats.

Parameters	Dose (mg/kg/day)	0	15	30	60
	No. of rats, M/F	10/10	10/10	10/10	10/10
*Home cage observations*					
Posture					
Sitting or standing alert		10/10	10/10	10/10	10/10
Abnormal vocalizations					
Absent		10/10	10/10	10/10	10/10
Convulsions					
Clonic and tonic movement					
Absent		10/10	10/10	10/10	10/10

*Open field observations*					
Gait					
Normal gait		10/10	10/10	10/10	10/10
Mobility score					
No impairment		10/10	10/10	10/10	10/10
Arousal level					
Alert		10/10	10/10	10/10	10/10
Clonic movement					
Absent		10/10	10/10	10/10	10/10
Tonic movement					
Absent		10/10	10/10	10/10	10/10
Stereotypic behavior					
Absent		10/10	10/10	10/10	10/10
Bizarre behavior					
Absent		10/10	10/10	10/10	10/10
Urination					
Normal		10/10	10/10	10/10	10/10
Defecation					
Normal		10/10	10/10	10/10	10/10
Rears (actual number)-males		66	61	73	60
Rears (actual number)-females		72	64	69	70
Vocalization					
Absent		10/10	10/10	10/10	10/10

*Sensory reactivity measurements*					
Approach response, touch response, click response, tail-pinch response, pupil response, aerial righting reflex					
Normal		10/10	10/10	10/10	10/10

**Table 3:** Hind limb foot splay, grip performance and motor activity of rats treated with SEM.

Parameters		Dose (mg/kg bwt/day)			
		0	15	30	60
Males					
Hind limb foot splay (mm)		65.21 ± 13.14	68.52 ± 8.26	59.58 ± 8.14	66.83 ± 7.26
Grip performance (kg)	Fore limb	1.63 ± 0.19	1.67 ± 0.08	1.61 ± 0.16	1.63 ± 0.14
	Hind limb	0.86 ± 0.07	0.91 ± 0.06	0.85 ± 0.09	0.88 ± 0.09
Motor activity	Total stereotypic time (sec)	753.73 ± 130.12	784.38 ± 148.06	822.76 ± 896.26	766.1 ± 948.33
	Total ambulatory time (sec)	993.28 ± 143.21	967.59 ± 176.27	985.27 ± 896.26	990.35 ± 836.28
	Total horizontal counts	4953.61 ± 136.36	4866.06 ± 238.37	5033.28 ± 896.26	5149.74 ± 1033.45
	Total ambulatory counts	3127.11 ± 151.93	3355.62 ± 227.27	3000.81 ± 896.26	3546.88 ± 947.37
Females					
Hind limb foot splay (mm)		63.84 ± 10.11	67.21 ± 11.26	64.22 ± 13.13	71.83 ± 7.48
Grip performance (kg)	Forelimb	1.31 ± 0.1	1.36 ± 0.17	1.39 ± 0.09	1.34 ± 0.12
	Hind limb	0.71 ± 0.07	0.74 ± 0.07	0.69 ± 0.03	0.73 ± 0.06
Motor activity	Total stereotypic time (sec)	1062.36 ± 75.36	1124.52 ± 151.37	993.82 ± 1136.37	1151.29 ± 631.37
	Total ambulatory time (sec)	1226.43 ± 100.36	1159.37 ± 210.74	1128.38 ± 1763.28	1213.22 ± 1381.29
	Total horizontal counts	9854.2 ± 137.37	8931.26 ± 183.29	8258.71 ± 1888.29	7900.65 ± 1138.83
	Total ambulatory counts	7036.58 ± 70.62	6837.04 ± 99.31	7364.55 ± 658.38	6538.75 ± 638.22

Values are expressed as mean ± SD of ten animals in each group.

**Table 4:** Length (days) of estrous cyclicity.

Dose (mg/kg bwt/day)	Estrous cyclicity length (days)
0	4.76 ± 0.74
15	4.43 ± 0.58
30	4.58 ± 0.47
60	4.32 ± 0.63

Values are expressed as mean ± SD of ten animals in each group.

**Table 5:** Postnatal developmental observation in pups: balano-preputial separation and vaginal opening.

Dose (mg/kg bwt/day)	Mean days of balano-preputial separation	Body weight (g) at the time of balano-preputial separation	Mean days of the vaginal opening	Body weight (g) at the time of vaginal opening
0	45.98 ± 1.07	159.63 ± 4.61	36.21 ± 1.26	91.22 ± 8.21
15	46.88 ± 2.11	171.27 ± 13.22	36.89 ± 1.13	91.68 ± 3.31
30	47.63 ± 1.63^*∗*^	162.83 ± 22.14	37.27 ± 1.2	93.47 ± 6.27
60	48.28 ± 1.94^*∗*^	175.38 ± 11.27^*∗*^	39.11 ± 1.13^*∗*^	96.37 ± 4.27

Values are expressed as mean ± SD of ten animals in each group. ^*∗*^Significantly different from the control, *p* ≤ 0.05.

**Table 6:** Hematological parameters of animals treated with semicarbazide.

Parameters	0 mg/kg	15 mg/kg	30 mg/kg	60 mg/kg
Males				
RBC (T/L)	9.86 ± 0.43	9.18 ± 0.12	9.28 ± 0.38	9.83 ± 0.41
Hb (g/L)	157.37 ± 4.81	161.22 ± 3.26	163.27 ± 3.75	161.26 ± 2.68
Hct (L/L)	0.482 ± 0.02	0.479 ± 0.01	0.481 ± 0.01	0.373 ± 0.02^*∗*^
MCV (fL)	49.52 ± 1.98	51.27 ± 1.47	53.53 ± 2.04	50.84 ± 1.94
	16.61 ± 0.51	16.37 ± 0.44	16.43 ± 0.56	16.52 ± 0.44
MCHC (g/L)	298.62 ± 5.85	293.71 ± 6.39	301.36 ± 5.27	295.27 ± 6.43
Retic (T/L)	0.21 ± 0.02	0.25 ± 0.02	0.23 ± 0.01	0.34 ± 0.02^*∗*^
Plat (G/L)	998.25 ± 183.29	983.19 ± 134.29	1007.65 ± 85.29	993.83 ± 97.61
MPV (fL)	8.81 ± 0.37	8.56 ± 0.68	8.73 ± 0.86	8.67 ± 0.73
WBC (G/L)	9.31 ± 2.73	9.27 ± 1.92	9.44 ± 1.83	10.11 ± 1.60
Neut (G/L)	1.48 ± 0.27	1.69 ± 0.35	1.51 ± 0.42	1.44 ± 0.53
Lymp (G/L)	7.33 ± 0.96	7.51 ± 0.73	8.30 ± 1.32	7.48 ± 0.58
Mono (G/L)	0.33 ± 0.12	0.38 ± 0.11	0.29 ± 0.18	0.31 ± 0.16
Baso (G/L)	0.04 ± 0.01	0.04 ± 0.01	0.04 ± 0.01	0.04 ± 0.02
Eosi (G/L)	0.09 ± 0.02	0.08 ± 0.03	0.08 ± 0.04	0.09 ± 0.07
Females				
RBC (T/L)	8.22 ± 0.27	8.51 ± 0.48	8.49 ± 0.37	8.20 ± 0.39
Hb (g/L)	146.27 ± 6.38	143.29 ± 5.83	147.52 ± 4.82	144.16 ± 4.50
Hct (L/L)	0.423 ± 0.01	0.442 ± 0.01	0.438 ± 0.01	0.441.0.01
MCV (fL)	60.13 ± 1.31	59.84 ± 1.22	63.27 ± 0.98	57.62 ± 1.04
MCH (pg)	17.83 ± 0.38	17.61 ± 0.44	17.51 ± 0.41	17.80 ± 0.37
MCHC (g/L)	299.38 ± 3.84	304.84 ± 4.73	291.04 ± 3.84	300.63 ± 3.93
Retic (T/L)	0.261 ± 0.04	0.278 ± 0.03	0.269 ± 0.04	0.294 ± 0.06^*∗*^
Plat (G/L)	1031.20 ± 153.31	1073.38 ± 160.36	1057.38 ± 143.27	1042.84 ± 164.39
MPV (fL)	8.83 ± 0.43	8.94 ± 0.51	8.57 ± 0.49	8.71 ± 0.52
WBC (G/L)	7.98 ± 1.28	7.83 ± 1.73	8.11 ± 1.64	7.29 ± 1.82
Neut (G/L)	0.86 ± 0.42	0.82 ± 0.37	0.81 ± 0.44	0.76 ± 0.53
Lymp (G/L)	7.01 ± 1.68	6.92 ± 1.39	6.93 ± 1.46	5.82 ± 1.50
Mono (G/L)	0.20 ± 0.05	0.18 ± 0.03	0.21 ± 0.15	0.17 ± 0.04
Baso (G/L)	0.03 ± 0.01	0.04 ± 0.01	0.03 ± 0.01	0.03 ± 0.01
Eosi (G/L)	0.08 ± 0.02	0.06 ± 0.04	0.07 ± 0.05	0.07 ± 0.04

Values are expressed as mean ± SD of ten animals in each group. ^*∗*^Significantly different from the control, *p* ≤ 0.05. RBC: red blood corpuscles; Hb: hemoglobin; Hct: hematocrit; MCV: mean corpuscular volume; MCH: mean corpuscular hemoglobin; MCHC: mean corpuscular hemoglobin concentration; Retic: reticulocytes count; Plat: platelets; MPV: mean platelet volume; WBC: white blood corpuscles; Neut: neutrophils; Lymp: lymphocytes; Mono: monocytes; Baso: basophils; Eosi: eosinophil.

**Table 7:** Clinical chemistry parameters of animals treated with semicarbazide.

Parameters	0 mg/kg	15 mg/kg	30 mg/kg	60 mg/kg
Males				
Glu (nmol/L)	7.91 ± 0.71	7.84 ± 0.65	7.81 ± 0.51	7.61 ± 0.76
BUN (nmol/L)	5.67 ± 0.58	5.23 ± 0.60	5.79 ± 0.85	5.45 ± 0.74
Creat (*µ*mol/L)	33.73 ± 6.29	27.92 ± 5.38	27.01 ± 4.50	30.03 ± 5.20
AST (U/L)	88.92 ± 37.01	81.17 ± 25.72	93.37 ± 43.28	102.01 ± 30.96^*∗*^
ALT (U/L)	67.23 ± 19.38	74.28 ± 35.24	78.27 ± 28.73	81.36 ± 30.35^*∗*^
GGT (U/L)	0.60 ± 0.82	0.61 ± 0.64	0.73 ± 0.58	0.70 ± 0.67
ALP (U/L)	127.74 ± 22.47	125.48 ± 19.46	128.40 ± 25.28	121.30 ± 17.31
LDH (U/L)	283.29 ± 192.28	359.21 ± 305.29	213.03 ± 246.33	296.22 ± 155.28
T. Bil (*µ*mol/L)	1.63 ± 0.73	1.28 ± 0.81	1.59 ± 0.45	1.89 ± 0.72
T. Chol (nmol/L)	3.41 ± 0.35	3.36 ± 0.28	3.28 ± 0.46	3.53 ± 0.39
Trig (nmol/L)	0.83 ± 0.17	0.88 ± 0.25	0.85 ± 0.37	0.88.29 ± 0.24
T. Pro (g/L)	73.21 ± 2.47	73.80 ± 2.59	76.00 ± 1.22	64.01 ± 2.38^*∗*^
Alb (g/L)	42.31 ± 1.38	44.28 ± 1.48	41.63 ± 1.56	47.23 ± 2.00
Glb (g/L)	27.82 ± 2.92	23.03 ± 2.93	26.64 ± 2.32	22.22 ± 2.06
A/G	1.71 ± 0.17	1.73 ± 0.13	1.75 ± 0.18	1.77 ± 0.20
Pi (nmol/L)	2.61 ± 0.11	2.63 ± 0.16	2.58 ± 0.14	2.60 ± 0.14
Ca (nmol/L)	2.78 ± 0.13	2.75 ± 0.16	2.63 ± 0.18	2.70 ± 0.15
Na (mEq/L)	137.72 ± 1.21	133.31 ± 1.04	135.92 ± 0.86	136.83 ± 1.41
K (mEq/L)	4.03 ± 0.36	4.11 ± 0.42	3.89 ± 0.37	4.21 ± 1.66
Cl (mEq/L)	83.52 ± 1.12	87.92 ± 1.24	85.30 ± 0.86	84.03 ± 1.25
Females				
Glu (nmol/L)	7.21 ± 0.67	7.35 ± 0.42	7.28 ± 0.53	7.40 ± 0.62
BUN (nmol/L)	5.21 ± 0.57	5.48 ± 0.63	5.33 ± 0.49	5.57 ± 0.37
Creat (*µ*mol/L)	29.88 ± 7.46	26.05 ± 8.28	22.21 ± 5.92	24.20 ± 4.83
AST (U/L)	97.83 ± 47.61	84.14 ± 10.20	92.94 ± 36.10	112.64 ± 63.12^*∗*^
ALT (U/L)	61.85 ± 26.22	66.39 ± 10.02	72.03 ± 15.21	84.55 ± 19.24^*∗*^
GGT (U/L)	0.77 ± 0.57	0.69 ± 0.49	0.72 ± 0.53	0.86 ± 0.84
ALP (U/L)	101.21 ± 16.33	98.20 ± 13.04	106.83 ± 11.52	96.22 ± 17.92
LDH (U/L)	194.43 ± 38.28	234.26 ± 29.47	217.04 ± 31.30	259.11 ± 53.39
T. Bil (*µ*mol/L)	1.81 ± 0.42	1.76 ± 0.39	1.66 ± 0.43	1.84 ± 0.51
T. Chol (nmol/L)	3.86 ± 0.31	3.73 ± 0.73	3.81 ± 0.36	3.63 ± 0.72
Trig (nmol/L)	0.58 ± 0.13	0.66 ± 0.11	0.71 ± 0.16	0.60 ± 0.15
T. Pro (g/L)	59.84 ± 2.23	66.30 ± 3.51	57.28 ± 2.10	55.52 ± 1.63
Alb (g/L)	44.29 ± 2.16	48.36 ± 1.38	40.61 ± 1.52	45.28 ± 1.94
Glb (g/L)	20.61 ± 1.47	21.52 ± 1.20	21.04 ± 2.00	20.61 ± 1.35
A/G	1.86 ± 0.13	1.81 ± 0.17	1.82 ± 0.11	1.76 ± 0.21
Pi (nmol/L)	2.02 ± 0.21	2.04 ± 0.26	2.16 ± 0.18	2.14 ± 0.20
Ca (nmol/L)	2.43 ± 0.11	2.40 ± 0.16	2.53 ± 0.14	2.47 ± 0.21
Na (mEq/L)	153.61 ± 1.73	157.74 ± 1.38	152.33 ± 0.94	154.39 ± 0.91
K (mEq/L)	3.56 ± 0.19	3.60 ± 0.21	3.52 ± 0.27	3.61 ± 0.18
Cl (mEq/L)	100.03 ± 1.10	104.46 ± 1.00	98.37 ± 2.04	101.46 ± 0.96

Values are expressed as mean ± SD of ten animals in each group. ^*∗*^: Significantly different from the control, *p* ≤ 0.05. Glu: glucose; BUN: blood urea nitrogen; Creat: creatinine; AST: aspartate aminotransferase; ALT: alanine aminotransferase; GGT: gamma glutamyl transpeptidase; ALP: alkaline phosphatase; LDH: lactate dehydrogenase; T.Bil: total bilirubin; T.Chol: total cholesterol; Trig: triglycerides; T. Pro: total plasma protein; Alb: albumin; Glb: globulin; A/G: albumin/globulin ratio; Pi: inorganic phosphorous; Ca: calcium; Na: sodium; K: potassium; Cl: chloride.

**Table 8:** Thyroid hormone analysis in male and female rats treated with SEM.

Parameters	Males				Females			
	0 mg/kg	15 mg/kg	30 mg/kg	60 mg/kg	0 mg/kg	15 mg/kg	30 mg/kg	60 mg/kg
TSH (ng/mL)	5.21 ± 3.64	4.58 ± 4.77	5.83 ± 6.33	4.29 ± 3.58	4.13 ± 2.82	3.25 ± 2.97	3.81 ± 1.47	2.98 ± 2.30
T4 (ng/mL)	46.34 ± 9.74	48.51 ± 12.94	42.10 ± 10.13	39.62 ± 9.21	24.81 ± 5.29	26.34 ± 7.41	21.20 ± 7.79	20.05 ± 10.36

Values are expressed as mean ± SD of ten animals in each group.

**Table 9:** Terminal fasting body weights, organ weights, and organ to body weight ratios of animals treated with semicarbazide.

Parameters		0 mg/kg	15 mg/kg	30 mg/kg	60 mg/kg
Males					
Terminal fasting body weight (g)		334.62 ± 11.31	320.53 ± 12.63	315.73 ± 20.26	337.30 ± 16.44
Adrenals	(mg)	60.0 ± 0.00	60.0 ± 0.00	60.0 ± 0.00	60.0 ± 0.00
	(%)	0.02 ± 0.00	0.02 ± 0.00	0.02 ± 0.00	0.02 ± 0.00
Brain	(g)	1.90 ± 0.07	1.92 ± 0.05	1.87 ± 0.04	1.86 ± 0.05
	(%)	0.54 ± 0.03	0.56 ± 0.03	0.58 ± 0.04	0.52 ± 0.02
Epididymides	(g)	1.24 ± 0.14	1.22 ± 0.11	1.27 ± 0.17	1.21 ± 0.13
	(%)	0.40 ± 0.03	0.44 ± 0.04	0.43 ± 0.05	0.40 ± 0.04
Heart	(g)	1.17 ± 0.14	1.16 ± 0.12	1.18 ± 0.17	1.15 ± 0.09
	(%)	0.29 ± 0.03	0.36 ± 0.01	0.31 ± 0.03	0.30 ± 0.04
Kidneys	(g)	1.83 ± 0.14	1.91 ± 0.13	1.86 ± 0.16	1.81 ± 0.13
	(%)	0.64 ± 0.04	0.65 ± 0.02	0.61 ± 0.05	0.64 ± 0.05
Liver	(g)	9.17 ± 0.58	8.83 ± 0.62	8.97 ± 0.71	11.25 ± 0.69^*∗*^
	(%)	2.91 ± 0.21	2.96 ± 0.26	3.11 ± 0.18	3.14 ± 0.20^*∗*^
Pituitary	(g)	0.01 ± 0.00	0.01 ± 0.00	0.01 ± 0.00	0.01 ± 0.00
	(%)	0.003 ± 0.00	0.003 ± 0.00	0.003 ± 0.00	0.003 ± 0.00
Prostate	(g)	0.83 ± 0.10	0.87 ± 0.15	0.85 ± 0.14	0.82 ± 0.12
	(%)	0.30 ± 0.03	0.30 ± 0.02	0.27 ± 0.04	0.29 ± 0.03
Seminal vesicles and coagulating glands	(g)	1.42 ± 0.14	1.45 ± 0.12	1.48 ± 0.12	1.51 ± 0.10
	(%)	0.47 ± 0.04	0.44 ± 0.04	0.42 ± 0.06	0.43 ± 0.05
Spleen	(g)	0.82 ± 0.06	0.87 ± 0.04	0.75 ± 0.07	0.90 ± 0.10^*∗*^
	(%)	0.27 ± 0.01	0.26 ± 0.02	0.23 ± 0.01	0.35 ± 0.04^*∗*^
Testes	(g)	3.61 ± 0.18	3.73 ± 0.21	3.70 ± 0.26	2.88 ± 0.22^*∗*^
	(%)	1.26 ± 0.07	1.28 ± 0.05	1.25 ± 0.07	1.13 ± 0.05^*∗*^
Thymus	(mg)	510.0 ± 0.08	480.0 ± 0.06	540.0 ± 0.06	400.0 ± 0.04^*∗*^
	(%)	0.17 ± 0.02	0.20 ± 0.01	0.17 ± 0.02	0.12 ± 0.01^*∗*^
Thyroid with parathyroids	(g)	0.03 ± 0.00	0.03 ± 0.00	0.03 ± 0.00	0.03 ± 0.00
	(%)	0.008 ± 0.00	0.008 ± 0.00	0.006 ± 0.00	0.007 ± 0.00

Values are expressed as mean ± SD of ten animals in each group. ^*∗*^Significantly different from the control, *p* ≤ 0.05.

**Table 10:** Terminal fasting body weights, organ weights, and organ to body weight ratios of animals treated with semicarbazide.

Parameters		0 mg/kg	15 mg/kg	30 mg/kg	60 mg/kg
Females					
Terminal fasting body weight (g)		207.26 ± 8.83	200.33 ± 16.84	209.24 ± 12.70	214.59 ± 11.37
Adrenals	(mg)	60.0 ± 0.00	60.0 ± 0.00	60.0 ± 0.00	60.0 ± 0.00
	(%)	0.02 ± 0.00	0.02 ± 0.00	0.02 ± 0.00	0.02 ± 0.00
Brain	(g)	1.81 ± 0.05	1.83 ± 0.02	1.80 ± 0.06	1.81 ± 0.03
	(%)	0.74 ± 0.03	0.72 ± 0.05	0.76 ± 0.05	0.75 ± 0.03
Heart	(g)	0.83 ± 0.04	0.80 ± 0.03	0.87 ± 0.07	0.82 ± 0.06
	(%)	0.42 ± 0.04	0.42 ± 0.03	0.47 ± 0.07	0.45 ± 0.04
Kidneys	(g)	1.42 ± 0.13	1.38 ± 0.09	1.45 ± 0.16	1.41 ± 0.08
	(%)	0.56 ± 0.03	0.58 ± 0.02	0.55 ± 0.04	0.57 ± 0.03
Liver	(g)	5.17 ± 0.71	5.19 ± 0.63	5.24 ± 0.50	5.96 ± 0.47^*∗*^
	(%)	2.58 ± 0.29	2.61 ± 0.35	2.66 ± 0.42	3.13 ± 0.39^*∗*^
Ovaries	(g)	0.07 ± 0.01	0.07 ± 0.01	0.07 ± 0.02	0.08 ± 0.01
	(%)	0.03 ± 0.00	0.03 ± 0.00	0.03 ± 0.00	0.03 ± 0.00
Pituitary	(g)	0.01 ± 0.00	0.01 ± 0.00	0.01 ± 0.00	0.01 ± 0.00
	(%)	0.004 ± 0.00	0.004 ± 0.00	0.004 ± 0.00	0.004 ± 0.00
Spleen	(g)	0.63 ± 0.04	0.61 ± 0.02	0.63 ± 0.06	0.77 ± 0.04^*∗*^
	(%)	0.21 ± 0.01	0.23 ± 0.02	0.20 ± 0.01	0.29 ± 0.02^*∗*^
Thymus	(mg)	290.0 ± 0.02	290.0 ± 0.04	270.0 ± 0.02	290.0 ± 0.03
	(%)	0.14 ± 0.01	0.13 ± 0.01	0.14 ± 0.01	0.11 ± 0.01
Thyroid with parathyroids	(g)	0.02 ± 0.00	0.02 ± 0.00	0.02 ± 0.00	0.02 ± 0.00
	(%)	0.01 ± 0.00	0.01 ± 0.00	0.01 ± 0.00	0.01 ± 0.00
Uterus with cervix (g)	(g)	0.68 ± 0.27	0.71 ± 0.43	0.63 ± 0.51	0.63 ± 0.66
	(%)	0.31 ± 0.17	0.30 ± 0.14	0.34 ± 0.18	0.30 ± 0.11

Values are expressed as mean ± SD of ten animals in each group. ^*∗*^Significantly different from the control, *p* ≤ 0.05.

**Table 11:** Vas deferens sperm motility, sperm morphology, and cauda epididymis sperm count of rats treated with SEM.

Dose (mg/kg bwt/day)	Motility		Morphology		Cauda epididymal sperm count		
	Percentage of progressively motile sperms	Percentage of motile sperms	Percentage of normal sperms	Percentage of abnormal sperms	Cauda epididymis weight (g)	No. of sperms per cauda epididymis (x 106)	No. of sperms per gram of cauda epididymis (x 106)
0	71.3 ± 7.82	78.5 ± 4.29	98.2 ± 0.67	0.22 ± 0.58	0.197 ± 0.02	197.28 ± 16.72	767.32 ± 44.27
15	72.4 ± 6.44	80.2 ± 6.28	98.0 ± 1.22	0.36 ± 1.08	0.193 ± 0.01	194.23 ± 13.53	760.29 ± 28.48
30	72.8 ± 5.84	79.7 ± 5.25	95.5 ± 1.13	1.11 ± 1.0	0.207 ± 0.03	195.85 ± 33.4	764.04 ± 34.91
60	63.6 ± 6.39^*∗*^	70.3 ± 4.12^*∗*^	85.3 ± 2.27^*∗*^	4.81 ± 2.21^*∗*^	0.211 ± 0.02	174.92 ± 27.39	736.31 ± 36.16

Values are expressed as mean ± SD of ten animals in each group. ^*∗*^Significantly different from the control, *p* ≤ 0.05.

**Table 12:** Summary of histopathological findings.

Sex		Males				Females			
Tissue and observation	Group no.	G1	G2	G3	G4	G1	G2	G3	G4
	Dose (mg/kg bwt/day)	0	15	30	60	0	15	30	60
	No. of rats	10	10	10	10	10	10	10	10
	No. of rats examined	10	10	10	10	10	10	10	10
1	Spleen	(10)	(10)	(10)	(10)	(10)	(10)	(10)	(10)
	Extramedullary hematopoiesis	1	—	2	3	—	—	—	—
2	Adrenal	(10)	(10)	(10)	(10)	(10)	(10)	(10)	(10)
	Cortical hypertrophy	1	—	1	5	0	2	2	3
3	Thymus	(10)	(10)	(10)	(10)	(10)	(10)	(10)	(10)
	Lymphoid depletion	2	1	3	2	—	—	1	4
4	Liver	(10)	(10)	(10)	(10)	(10)	(10)	(10)	(10)
	Centrilobular hepatocyte hypertrophy	1	2	2	5	—	2	1	3

## Data Availability

The data used to support the findings of this study are available from the corresponding author upon request.
